# Phytochemical Composition, Antioxidant Activity, and Neuroprotective Effect of *Terminalia chebula* Retzius Extracts

**DOI:** 10.1155/2012/125247

**Published:** 2011-07-05

**Authors:** Chia Lin Chang, Che San Lin

**Affiliations:** Research Institute of Biotechnology, HungKuang University, 34 Chung-Chie Rd, Sha Lu, Taichung 43302, Taiwan

## Abstract

The objectives of this study were to determine phytochemical compositions, chemiluminescence antioxidant activities, and neuroprotective effects on PC12 cells for water, methanol, and 95% ethanol extracts of the air-dried fruit of *Terminalia chebula* Retzius. The water extract afforded the greatest yield, and total phenolic and tannin content. The methanol extract yielded the greatest total triterpenoid content. Based on four chemiluminescence antioxidant assays, the three extracts showed various degrees of antioxidant activity. The methanol extract showed good antioxidant activity based on the horseradish peroxidase-luminol-hydrogen peroxide (H_2_O_2_) assay. The water extract appeared to have good antioxidant activities in cupric sulfate-Phen-Vc-H_2_O_2_ and luminol-H_2_O_2_ assays. Pyrogallol-luminol assay showed the 95% ethanol extract to have good antioxidant activity. The methanol and water extracts presented neuroprotective activities on H_2_O_2_-induced PC12 cell death at 0.5–5.0 *μ*g/mL. Further investigations are necessary to verify these activities *in vivo*.

## 1. Introduction

The auto-oxidation of lipids generates reactive oxygen species (ROS) such as superoxide anion radicals (^∙^O_2_^−^), hydroxyl radicals (^•^OH), and hydrogen peroxide (H_2_O_2_) [1]. Excessive production of ROS is implicated in ageing as well as in many diseases, including atherosclerosis, cancer, and inflammatory disease [2]. Antioxidants are very important for human health, and thus antioxidant supplementation is recommended to provide cellular protection from the deleterious effects of excessive ROS concentrations [3]. In this study, we applied chemiluminescence techniques to assay three *Terminalia chebula* Retzius extracts for antioxidant activity. Chemiluminescence antioxidant assay is a simple, direct, and effective method well suited to free radicals and antioxidant study [4].

*T. chebula* is a traditional medicine belonging to the genus Terminalia, family Combretaceae, and is extensively cultivated in Taiwan. The dried ripe fruit of* T. chebula* is an important Indian herb used extensively in the indigenous system of medicine (ayurvedic) for its homeostatic, antitussive, laxative, diuretic, and cardiotonic activities [5]. It also exhibits the ability to scavenge the 1,1-diphenyl-2-picrylhydrazyl radicals [6–8]. Phytochemical analysis of *T. chebula* shows the presence of gallic acid, ellagic acid, tannic acid, ethyl gallate, chebulic acid, chebulagic acid, corilagin, mannitol, ascorbic acid (vitamin C), and other compounds [9]. One source lists *T. chebula* as having 32% tannin content [10]. Thus, phytochemical analyses of *T. chebula* extract composition are necessary and provide useful information.

The nature of the extracting solvent is the most important factor in the extraction of antioxidants [11]. Polar solvents and alcoholic solutions frequently provide satisfactory extraction, and the most suitable for plant extractions are methanol, water, and ethanol [12, 13]. For the plant fruit extractions in this study, we used ultrasonic extraction with water, methanol, and 95% ethanol in preference to other solvent choices. We conducted phytochemical composition analyses for total phenolic, triterpenoid, and tannin content. Four chemiluminescence antioxidant methods, based on scavenging of the luminol radicals, ^∙^O_2_^−^, ^•^OH, and H_2_O_2_, were used to analyze the three *T. chebula* extracts. The rat pheochromocytoma cell line (PC12, CRL-1721) is a useful model in the study of neurodegenerative disease and the neuroprotective effects [14]. We previously reported that *T. chebula* extracts stimulate PC12 cell growth [15]. Therefore, we also researched the neuroprotective effects of the three extracts on H_2_O_2_-induced PC12 cell death.

## 2. Materials and Methods

### 2.1. Materials

*T. chebula* was obtained from the air-dried fruit, purchased from Xin Long Pharmaceutical Limited Company (Taichung, Taiwan). Methanol (Mallinckrodt Baker, Inc., Phillipsburg, U.S.A.) and 95% ethanol (Uni-Onward Corporation, Taipei, Taiwan) were both purchased as ACS grade reagents. Deionized water was obtained from an Ultrapure Water System (Putity-UV, Suntex Instruments Corporation, LTD., Taipei, Taiwan). Phosphate buffer (0.1 M, pH 7.4) was prepared from sodium phosphate monobasic monohydrate and sodium phosphate dibasic dodecahydrate. Sodium phosphate monobasic monohydrate, sodium phosphate dibasic dodecahydrate, boric acid, gallic acid, glacial acetic acid, perchloric acid, vitamin C, luminol, horseradish peroxidase (HRP), pyrogallol, sodium bicarbonate (NaHCO_3_), vanillin, L-ascorbic acid sodium, 3-(4,5-dimethyl-2-thiazolyl)-2,5-diphenyl-2H-tetrazolium bromide (MTT), poly-L-lysine hydrobromide, N-acetyl-Asp-Glu-Val-Asp-al (AC-DEVD-CHO), sodium carbonate (Na_2_CO_3_), ethylenediaminetetraacetic acid (EDTA), cupric sulfate (CuSO_4_), 6-hydroxy-2,5,7,8-tetramethylchroman-2-carboxylic acid (trolox), zinc acetate (ZnAc), 1,10-phenanthroline, ammonia (NH_3_), and ammonium chloride (NH_4_Cl) were originally obtained from Sigma-Aldrich Corporation, Shanghai, China. Trolox and vitamin C were used as positive control samples over an optimized concentration range. Trolox is the hydrophilic analog of vitamin E, and vitamin E is a fat soluble antioxidant. Vitamin C is a water soluble antioxidant. Folin-Ciocalteau reagent and ursolic acid were purchased from Fluka Biochemica (Buchs, Switzerland). Thirty-five percent H_2_O_2_ was purchased from Riedel-de Haën (Seelze, Germany). Dimethyl sulphoxide (DMSO) and ethanol were purchased from Merck (Darmstadt, Germany). Dr. Y. C. Shen, of the National Research Institute of Chinese Medicine (Taipei, Taiwan), kindly provided the PC12 cells. Dulbecco's modified Eagle's medium (DMEM), heat-inactivated horse serum (HS), heat-inactivated fetal bovine serum (FBS), penicillin/streptomycin, and L-glutamine were purchased from HyClone (Tseng Hsiang Life Science LTD., Taipei, Taiwan). The 100 mm cell culture dish was purchased from Greiner Bio-One (Bio-Check Laboratories LTD., Taichung, Taiwan).

### 2.2. Methanol, Water, and 95% Ethanol Extraction

*T. chebula* was pulverized into fine powder using a stainless steel blender (Waring Commercial, Torrington, Conn, U.S.A.). Two-gram aliquots of the dried powder were each extracted three times with methanol (20 mL), deionized water (20 mL), and 95% ethanol (20 mL). The mixtures were agitated in an ultrasonic cleaner (model DC200H, Chemist Scientific Corporation, Taipei, Taiwan) for 15 min at room temperature then filtered. The methanol, deionized water, and 95% ethanol filtrates were individually pooled and each solvent removed at 40°C, under reduced pressure by rotary evaporator (Rotavapor R210, Buchi, Postfach, Flawil, Switzerland). Finally, each extract was dried overnight in a freeze dryer (model FD3-12P-80°C, Kingmech Corporation, Taipei, Taiwan) before calculating the yield of each extract. All of the dried extracts were brown solids and were stored at −20°C prior to phytochemical composition analyses and bioassays.

### 2.3. Total Phenolic Content

The total phenolic contents of the three extracts were determined using the Folin-Ciocalteu assay [16]. Briefly, 0.6 mg samples of each of the three extracts were dissolved into methanol (1 mL), deionized water (1 mL), and 95% ethanol (1 mL), respectively, and then 11.4 *μ*L aliquots of each of these solutions were mixed with Na_2_CO_3_ (2%, 227.32 *μ*L). The mixtures were stood at room temperature for 2 min before the addition of Folin-Ciocalteau reagent (50%, 11.4 *μ*L) to each sample mixture. After incubation at room temperature for 30 min, the absorbances of the reaction mixtures were measured at 750 nm using a tunable microplate reader (VersaMax, Molecular Devices Corporation, Sunnyvale, Calif, U.S.A.). Gallic acid (0.2–1.0 mg/mL in methanol) was used as a standard, and the total phenolic contents of three extracts were expressed in milligram gallic acid equivalents (mg gallic acid/g extract).

### 2.4. Total Triterpenoid Content

After optimizing all experimental parameters, total triterpenoid content was determined by colorimetry using the following procedure [17]. Briefly, 10 mg of each of the three extracts was individually dissolved in 1 mL of methanol, deionized water, and 95% ethanol. Then, 100 *μ*L of each of these solutions was mixed with vanillin/glacial acetic acid (150 *μ*L, 5% w/v) and perchloric acid solution (500 *μ*L). The sample solutions were heated for 45 min at 60°C and then cooled in an ice-water bath to the ambient temperature. After the addition of glacial acetic acid (2.25 mL), each sample solution's absorbance was measured at 548 nm, using a UV-visible-light recording spectrophotometer (UV-160 A; Shimadzu Corporation, Kyoto, Japan). Ursolic acid (0.025–0.5 mg/mL in methanol) was used as a standard. Results were expressed as milligram ursolic acid equivalents (mg ursolic acid/g extract).

### 2.5. Total Tannin Content

Analysis of total tannin was based on a titrimetric method [18]. Zinc ion reacts with tannin compounds in alkali solution, to form complexes. Residual zinc ion is then titrated with EDTA, and zinc complexed tannin is determined from EDTA consumption and total zinc content. The methanol, water, and 95% ethanol extracts (1 mg each) were each placed into glass vials and dissolved with 1 mL of deionized water. The vials were warmed in a water bath for 5 min at 35 ± 2°C. ZnAc (1 M, 0.4 mL) and NH_3_ (0.28 mL) were mixed together, and the warmed 1-mL extract solutions were added. The solutions were replaced in the water bath for 30 min at 35 ± 2°C. Deionized water (8.92 mL) was added to make the final volume up to 10.6 mL. After careful filtering, sample solutions were obtained. The solutions (0.8 mL) were further diluted with 5.2 mL of deionized water, and 0.5 mL of NH_3_–NH_4_Cl buffer (pH 10) was added. Finally, the mixture was titrated with 0.05 M EDTA. The blank was detected without addition of the extract. The total tannin content (%/mg extract) of each extract was calculated as follows: {[0.1556 × (*V*_blank_ − *V*_extract_)]/*W*_extract_} × 100%. *V*_blank_ and *V*_extract_ represent the EDTA titration volumes (mL) recorded for the respective blank and extract solutions. *W*_extract_ represents the weight (mg) of each extract [19].

### 2.6. HRP-Luminol-H_2_O_2_ System

Samples of 2.5 *μ*L of trolox, vitamin C, *T. chebula* extracts, and DMSO as control were prepared with three replicates, to concentrations of 2.0, 3.9, 7.8, 15.6, 31.3, 62.5, 125.0, 250.0, 500.0, and 1000.0 *μ*g/mL. The samples were mixed with 238.8 *μ*L phosphate buffer (0.1 M, pH 7.4), and 2.6 *μ*L luminol solution (20 mg/mL in DMSO) was added to yield a final luminol concentration of 1.16 × 10^−3^ M. H_2_O_2_ (1.1 *μ*L) was then added to bring the final concentration to 5 × 10^−2^ M H_2_O_2_. HRP (5.0 *μ*L, 10 IU/mL) was then added to a concentration of 0.2 IU/mL HRP and a total solution volume of 250 *μ*L. Chemiluminescence was measured with a microplate luminometer apparatus (LUMIstar OPTIMA, BMG LABTECH GmbH, Offenburg, Germany) for 40 min at 25°C. Integration of the chemiluminescence time-course curves provided an estimate of each sample's relative inhibitory activity under various concentrations. The inhibition ratio (%) of each sample is calculated as follows: [1 − (AUC_sample_/AUC_control_)] × 100%. Where AUC_sample_ and AUC_control_ represent the area under the time-course curve measured for the sample solution and control, respectively [20].

### 2.7. Pyrogallol-Luminol System

Samples of 2.5 *μ*L of trolox, vitamin C, *T. chebula* extracts, and DMSO as control were prepared with three replicates, to concentrations of 3.9, 7.8, 15.6, 31.3, 62.5, 125.0, 250.0, 500.0, and 1000.0 *μ*g/mL. The samples were mixed with pyrogallol (6.25 × 10^−4^ M, 12.5 *μ*L), and 235 *μ*L of a mixture containing Na_2_CO_3_–NaHCO_3_ buffer (0.05 M, pH 10.2) with 0.1 mM EDTA/1 mM luminol (2 : 1 v : v) solution was added to yield a final volume of 250 *μ*L. The background was detected without the addition of pyrogallol solution. Chemiluminescence was measured for 10 min at 25°C with a microplate luminometer. Integration of the sample chemiluminescence time-course curves provided the relative scavenging activity of each sample, estimated at various concentrations. The scavenging activity ratio (%) of each sample was calculated as follows: {[(AUC_control_ − AUC_background_) − (AUC_sample_ − AUC_background_)]/[AUC_control_ − AUC_background_]} × 100%. Where AUC_control_, AUC_background_, and AUC_sample_ represent the area under the time-course curve measured for the control, background, and sample, respectively [21].

### 2.8. CuSO_4_-Phen-Vc-H_2_O_2_ System

Samples of 12.5 *μ*L of trolox, vitamin C, *T. chebula* extracts, and DMSO as control were prepared with three replicates, to concentrations of 3.9, 7.8, 15.6, 31.3, 62.5, 125.0, 250.0, 500.0, and 1000.0 *μ*g/mL. The samples were mixed with CuSO_4_ solution (1.0 mM, 12.5 *μ*L), 1,10-phenanthroline solution (1.0 mM, 12.5 *μ*L), borate buffer (0.05 M, 175.0 *μ*L, pH 9.0), and L-ascorbate solution (1.0 mM, 25.0 *μ*L). Additionally, H_2_O_2_ solution (12.5 *μ*L, 0.15% v/v) was added to yield a final volume of 237.5 *μ*L. The background was detected without the addition of H_2_O_2_ solution. Chemiluminescence was measured for 10 min at 25°C with a microplate luminometer. The scavenging activity ratio (%) of each sample was calculated using the formula described for the pyrogallol-luminol system [21].

### 2.9. Luminol-H_2_O_2_ System

Samples of 12.5 *μ*L of trolox, vitamin C, *T. chebula* extracts, and DMSO as control were prepared with three replicates, to concentrations of 3.9, 7.8, 15.6, 31.3, 62.5, 125.0, 250.0, 500.0, and 1000.0 *μ*g/mL. The samples were mixed with H_2_O_2_ solution (0.15 M, 12.5 *μ*L). A solution containing Na_2_CO_3_–NaHCO_3_ buffer solution (225 *μ*L, 0.05 M, pH 9.4) and luminol solution (0.1 mM, 17 : 1 v: v) was added to yield a final volume of 250.0 *μ*L. The background was detected without the addition of H_2_O_2_ solution. Chemiluminescence was measured for 40 min at 25°C with a microplate luminometer apparatus. The scavenging activity ratio (%) of each sample was calculated using the formula described for the pyrogallol-luminol system [21].

### 2.10. Protective Effect

PC12 cells were plated on poly-L-lysine hydrobromide-coated 100 mm cell culture dishes and grown in DMEM, supplemented with 10% HS, 1% FBS, a mixture of 1% penicillin/streptomycin, and 1% L-glutamine at 37°C in a 95% humidified air-5% CO_2_ chamber. Cells were subcultured for up to ten passages. Cellular viability was determined using the trypan blue exclusion test. Only cell preparations with 95% or greater viability were used. PC12 cells were seeded in poly-L-lysine hydrobromide-coated 24-well cell culture plates (1.25 × 10^5^ cells/well) with complete DMEM for 24 h. H_2_O_2_ was added to induce PC12 cell death. To study the protective effect of test samples on the PC12 cells, we renewed the media and preincubated the cells for 12 h, either with or without the presence of 500 *μ*L test samples, to obtain sample concentrations of 0, 0.5, 2.5, and 5.0 *μ*g/mL. Thereafter, the media was replaced, and 500 *μ*L H_2_O_2_ was added to a concentration of 40 *μ*M H_2_O_2_, and the mixture was incubated for an additional 12 h. The control was incubated without the addition of either sample or H_2_O_2_ solution.

All extracts were dissolved in DMSO. The concentration of DMSO in the final culture medium was 0.5%, which had no observable effect on cell viability as determined by MTT reduction assay, following Choi et al.'s method [22] with slight modifications. Upon completion of incubation, MTT solution (50 *μ*L, 1.0 mg/mL) was added to the culture medium, and the cells were incubated for 2 h at 37°C. The medium was then removed, and 300 *μ*L of DMSO was added to the well to dissolve the formazan, derived from live cell mitochondrial cleavage of the tetrazolium ring. The formazan solutions were incubated for 30 min at 25°C, and formazan solutions (250 *μ*L) were placed in 96-well plates. The amount of MTT formazan product was determined by measuring optical density (OD) with a tunable microplate reader at a test wavelength of 570 nm and a reference wavelength of 655 nm. MTT reduction was calculated as OD_570 nm_ − OD_655 nm_. Cell viability (%) was determined as (OD_sample_/OD_control_) × 100%. Where OD_sample_ represents OD_sample  (570 nm)_ − OD_sample  (655 nm),_ and OD_control_ represents the mean value of OD_control  (570 nm)_ − OD_control  (655 nm)_. The control cells exhibited 100% cell viability. Gallic acid and AC-DEVD-CHO were the positive controls. Gallic acid exists in *T. chebula*, and AC-DEVD-CHO is a caspase 3 inhibitor.

### 2.11. Statistical Analysis

The IC_50_ value (the concentration of a sample that is required for 50% inhibition *in vitro*) was determined using linear regression. Each phytochemical characteristic and chemiluminescence antioxidant activity was determined three times, using the same extract in order to determine reproducibility and to provide a mean ± standard deviation (SD) using Microsoft Excel 2003. Cell viability was measured by MTT reduction assay. Cell viability (%) represents three replicates per treatment. For each sample's “protective effect” against H_2_O_2_ effects on PC12 cells, only data concerning PC12 cell group exposure to H_2_O_2_ was considered. All data were represented as means ± SD based on triplicate determinations. Data were analyzed for statistical significance using one-way ANOVA, followed by Tukey's test as a post-hoc test with SPSS software (SPSS for Windows, Version 10).

## 3. Results and Discussion

### 3.1. Extraction Yield, and Total Phenolic, Triterpenoid, and Tannin Content of Methanol, Water, and 95% Ethanol Extracts

Table 1 presents the yield, and total phenolic, triterpenoid, and tannin content of the three extracts. The yield of the three extracts varied from 21.7% to 39.4%. The polarity (*δP*) of methanol, water, and ethanol lays in the following descending order: water (16.0) > methanol (12.3) > ethanol (8.8). The water extract had the greatest yield of the three extracts. The lowest extraction yield of the 95% ethanol extract may be due to the high *δP* of the major components of *T. chebula*.

Total phenolic content in the three extracts was determined from a linear gallic acid standard curve. The total phenolic content of the three extracts varied from 867.2 to 1041.8 mg gallic acid/g extract. This result suggests that the water extract provided the greatest concentration of phenolic compounds of the three extracts. The total triterpenoid content of the three extracts was evaluated by colorimetry, using ursolic acid as the standard. The total triterpenoid content of the three extracts varied widely from 0.8 to 4.2 mg ursolic acid/g extract. The lowest total triterpenoid content occurred for the water extract, whereas the methanol extract provided the greatest triterpenoid content of the group. The triterpenoid is a low polarity compound, and nine oleanane-type triterpenoids were isolated from the methanol extract of *T. chebula* [23]. The total tannin content of the three extracts varied from 33.9 to 40.3%/mg extract. The greatest total tannin content was detected in the water extract. Finally, these results show that the water extract produced a higher total phenolic and tannin content than the methanol and 95% ethanol extracts did. This may be the cause of the best extraction yield seen for the *T. chebula* water extract.

### 3.2. Antioxidant Activity of the HRP-Luminol-H_2_O_2_ System

The antioxidant activity of three extracts was evaluated using a chemiluminescence assay method (Table 2). The scavenging effect of luminol radicals was observed upon addition of the three extracts. The antioxidant IC_50_ values for the three extracts ranged from 6.9 to 42.4 *μ*g/mL. The antioxidant abilities of the three extracts compared with vitamin C and trolox were in the order trolox > methanol extract > vitamin C > 95% ethanol extract > water extract. However, other workers using a different chemiluminescence method reported the scavenging activity of luminol radicals by the *T. chebula* water extract to be strong [24]. This finding shows that the high antioxidant activity observed for the methanol extract is a consequence of its total triterpenoid content.

### 3.3. Antioxidant Activity of the Pyrogallol-Luminol System

Pyrogallol was autoxidized under the alkaline condition to generate the ^∙^O_2_^−^ radical ion, and the scavenging effect of this radical was observed on addition of each of the three extracts. The IC_50_ values for the three extracts' antioxidant activity ranged from 146.0 to 235.5 *μ*g/mL (Table 2). The antioxidant properties of the three extracts compared with vitamin C and trolox are in the descending order of 95% ethanol extract > methanol extract > trolox > water extract > vitamin C. Another research group, using a different method [6, 25], reported the IC_50_ value of the ^∙^O_2_^−^ radical ion's scavenging activity for 70% methanol, methanol, and water extracts of *T. chebula* as 13.4, 480.0, and 730.0 *μ*g/mL, respectively. These findings show that each of the three extracts exhibit different antioxidant abilities. The greater scavenging effect of the ^∙^O_2_^−^ radical ion by the 95% ethanol extract relates to the extract's total triterpenoid content. The good ^∙^O_2_^−^ scavenging effect of the methanol extract results from the total triterpenoid and tannin content.

### 3.4. Antioxidant Activity of the CuSO_4_-Phen-Vc-H_2_O_2_ System

The ^•^OH scavenging effect was observed by adding each of the three extracts. The IC_50_ values for the three extract's antioxidant activity ranged from 4.5 to 6.7 *μ*g/mL (Table 2). The antioxidant properties of the three extracts compared with vitamin C and trolox are in the decreasing order of water extract > trolox > methanol extract > 95% ethanol extract > vitamin C. Compared to another published method, the IC_50_ value of the ^•^OH scavenging activity for 70% methanol extract of *T. chebula* was reported to be 72.0 *μ*g/mL [25]. This finding shows that the three extracts had different ^•^OH scavenging activities. The higher ^•^OH scavenging activity of the water extract is related to the amount of total phenolic and tannin content.

### 3.5. Antioxidant Activity of the Luminol-H_2_O_2_ System

In the oxygen and alkaline condition, H_2_O_2_ oxidizes luminol to produce luminescence. The IC_50_ values of the H_2_O_2_ scavenging activity for the three extracts ranged from 1.3 to 1.8 *μ*g/mL (Table 2). The antioxidant abilities of the three extracts, against both vitamin C and trolox, were in the order of trolox > water extract > methanol extract > 95% ethanol extract > vitamin C. Compared to another method, the IC_50_ value of the H_2_O_2_ scavenging activity for 70% methanol extract of* T. chebula* was found to be much higher than can be represented [25]. Thus, all three extracts were able to scavenge H_2_O_2_. The higher antioxidant activity of the water extract arises from the amount of total phenolic and tannin content.

### 3.6. Protective Effect

**Figure 1:**
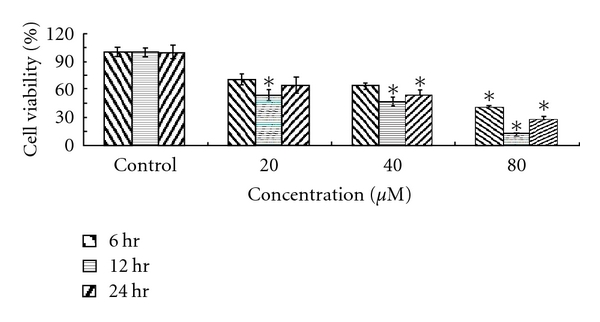
H_2_O_2_ induced a dose-dependent viability loss in PC12 cells. *P < .01 versus control (0 *μ*M).

In order to ascertain the neuroprotective effects of the *T. chebula* extracts, we applied an *in vitro* “Inhibition of H_2_O_2_-induced PC12 cell death” model to estimate the neuroprotective effect. Application of H_2_O_2_ induced a dose-dependent loss in viability of PC12 cells. The cell viability decreased to 12.2% when exposed to 80 *μ*M H_2_O_2_ for 12 h, as shown in Figure 1. In order to obtain a detectable effect, cells were treated with 40 *μ*M H_2_O_2_ for 12 h, causing cell viability to decrease to 47.3%.  Table 3 shows the results of this protective study; we found that cell viability decreased to 59.0 ± 5.1% when exposed to H_2_O_2_ (40 *μ*M) for 12 h. The methanol and water extracts inhibited H_2_O_2_-induced cytotoxicity at 0.5–5.0 *μ*g/mL. The water extract showed the greatest neuroprotective activity among the three extracts. The data of water extract is two times bigger than that of control because it alone did not show any cytotoxicity at 0.1–20.0 *μ*g/mL [15]. Meanwhile, the total time of this protective experiment was 24 h, and the water extract could induce cell proliferation at 0.5–5.0 *μ*g/mL for up to 72 h [15]. The result of the protective study did not show any cytotoxicity and neuroprotection when PC12 cells were pretreated with the water extract at 0.5–5.0 *μ*g/mL for 3 h prior to exposure to 40 *μ*M H_2_O_2_ for 12 h (data not shown). The short preincubation time (3 h) of the water extract did not induce PC12 cell proliferation and inhibit H_2_O_2_-induced cytotoxicity. While the 95% ethanol extract produced the greatest neurogrowth activity at 0.5–5.0 *μ*g/mL for up to 72 h [15], neither it, gallic acid, nor AC-DEVD-CHO provided any observable protection against H_2_O_2_ for the PC12 cells. Gallic acid induced the phosphorylation of c-Jun N-terminal protein kinase and the downregulation of Bcl-2 in PC12 cells. AC-DEVD-CHO did not protect against the microglia-induced PC12 cell death. Recent evidence also indicates that caspase 3 is not required for apoptosis in the PC12 cells. These present studies have clearly demonstrated that the pretreatments of the PC12 cells with the gallic acid and AC-DEVD-CHO did not reverse PC12 cell death [26–28]. Based on the results of this study and findings of our previous report [29], we propose that the effective neuroprotective activity of the water extract is a consequence of its ^•^OH and H_2_O_2_ scavenging activities, its greatest extraction yield, and its total phenolic and tannin content. These findings demonstrate that the methanol and water extracts of *T. chebula* reduce the rate of H_2_O_2_-induced PC12 cell death.

## 4. Conclusions

We demonstrated and compared for the first time the phytochemical compositions, chemiluminescence antioxidant activities, and neuroprotective effects of water, methanol, and 95% ethanol extracts of the air-dried fruit of *T. chebula* Retzius. The three extracts of *T. chebula* were the richest of phenolic compounds compared with total triterpenoid and tannin content as presented in Table 1. The methanol extract had the greatest total triterpenoid content and exhibited good antioxidant activity in the HRP-luminol-H_2_O_2_ assay. The water extract appeared to have the greatest total phenolic and tannin content and showed good antioxidant activities in both CuSO_4_-Phen-Vc-H_2_O_2_ and luminol-H_2_O_2_ assays. The 95% ethanol extract exhibited good antioxidant activity in the pyrogallol-luminol assay. Thus, the three extracts present various levels of ROS scavenging efficiency due to differences between the mechanisms of the four ROS chemiluminescence systems. The three extracts are new potential sources of natural antioxidants for food and nutraceutical products. The methanol and water extracts exhibit neuroprotective activities against H_2_O_2_-induced toxicity toward PC12 cells and are potential candidates for the treatment of H_2_O_2_-induced neurodegenerative disease. Further investigations are required to elucidate the exact mechanisms* in vivo* that these candidates operate by and to isolate and purify the major compound of each of the extracts.

## Figures and Tables

**Table 1:** Extraction yield, and total phenolic, triterpenoid, and tannin content of the three extracts for* Terminalia chebula*.

Extract	Extraction yield^a^	Total phenolic content^b^	Total triterpenoid content^c^	Total tannin content^d^
Methanol extract	24.2 ± 1.5	924.5 ± 17.2	4.2 ± 0.6	36.0 ± 4.6
Water extract	39.4 ± 1.8	1041.8 ± 8.6	0.8 ± 0.2	40.3 ± 2.3
95% ethanol extract	21.7 ± 1.1	867.2 ± 18.1	3.9 ± 0.5	33.9 ± 1.4

The data are presented as mean ± SD for three replicates.

^a^% w/w.

^b^mg gallic acid/g extract.

^c^mg ursolic acid/g extract.

^d^%/mg extract.

**Table 2:** Antioxidant activities of the three extracts for* Terminalia chebula* and positive control.

Extract and positive control	HRP-luminol-H_2_O_2_ assay^a^	Pyrogallol-luminol assay^a^	CuSO_4_-Phen-Vc-H_2_O_2_ assay^a^	Luminol-H_2_O_2_ assay^a^
Extract				
Methanol extract	6.9 ± 1.9	184.8 ± 12.3	6.3 ± 0.2	1.7 ± 0.3
Water extract	42.4 ± 4.1	235.5 ± 22.6	4.5 ± 1.2	1.3 ± 0.2
95% ethanol extract	17.7 ± 5.8	146.0 ± 5.9	6.7 ± 1.7	1.8 ± 0.2
Positive control				
Vitamin C	14.8 ± 6.2	>1000	688.3 ± 29.7	32.8 ± 8.9
Trolox	3.2 ± 0.1	209.6 ± 17.0	5.8 ± 1.0	0.8 ± 0.1

The data are presented as mean ± SD for three replicates.

^a^IC_50_ values in *μ*g/mL.

**Table 3:** Protective effects of the three extracts of *Terminalia chebula* and positive control on H_2_O_2_-induced PC12 cell death.

Sample	Concentration (*μ*g/mL)	Cell viability (%)
Control	0	100.0 ± 7.3

H_2_O_2_	40 *μ*M	59.0 ± 5.1^#^

Methanol extract	0.5	100.8 ± 9.2*
	2.5	107.1 ± 4.3*
	5.0	104.6 ± 4.3*

Water extract	0.5	174.3 ± 12.6*
	2.5	211.7 ± 11.7*
	5.0	287.0 ± 15.7*

95% ethanol extract	0.5	63.0 ± 4.4
	2.5	54.5 ± 8.3
	5.0	64.2 ± 6.0

Gallic acid	0.5	24.0 ± 4.2*
	2.5	29.0 ± 5.1*
	5.0	20.2 ± 5.2*

AC-DEVD-CHO	0.5	23.9 ± 4.2*
	2.5	19.7 ± 3.3*
	5.0	37.5 ± 5.8

The data are presented as mean ± SD for three replicates. ^#^ *P* < .01 versus the control group without the addition of the sample and H_2_O_2_ solutions, *P < .01 versus the 40 *μ*M H_2_O_2_-treated group without the addition of the sample solution.
